# Determination of Puberulic Acid in Monascus-Fermented Red Yeast Rice by LC-MS/MS Combined with Precolumn Derivatization

**DOI:** 10.3390/toxins17010011

**Published:** 2024-12-29

**Authors:** Hui-Qin Pan, Rui Feng, Yan-Nan Tan, Xiao-Ya Qin, Yi-Min Cao, Xiu-Hong Mao, Qing Hu, Heng Zhou

**Affiliations:** 1 NMPA Key Laboratory for Quality Control of Traditional Chinese Medicine, Shanghai Institute for Food and Drug Control, 1500 Zhangheng Road, Shanghai 201203, China; 2 School of Pharmacy, Shanghai University of Traditional Chinese Medicine, 1200 Cailun Road, Shanghai 201203, China

**Keywords:** red yeast rice, puberulic acid, LC-MS/MS, precolumn derivatization, methylation

## Abstract

Puberulic acid (PA) is a mycotoxin produced by a species of *Penicillium*. It has received widespread attention as a significant contributor to the reported fatalities associated with red yeast rice dietary supplements. However, the detection of PA, especially at low concentration levels, poses a considerable challenge, with no detection methods reported thus far. Here, we present a simple and sensitive derivatization-based LC-MS/MS method, requiring no purification processes, for determination of PA in the red yeast rice. The methylating derivatization with trimethylsilyldiazomethane (TMSCHN_2_) was performed to enhance its analytical performance. To achieve optimal detection sensitivity, the amount of solvent and TMSCHN_2_ for the derivatization reaction, along with the reaction time, were individually optimized. Moreover, sample extraction solvent was carefully chosen to improve recoveries in real sample analyses. Comparatively, the proposed LC-MS/MS method achieved a superior detection sensitivity, over 100-fold higher than that of the liquid chromatography method. A good linear relationship within the concentration range of 5 ng/mL to 200 ng/mL (with a linear correlation coefficient of 0.99952) was demonstrated by the method validation. The average recovery rate was between 82.2% and 84.2%, and the repeatability (RSD of 2.1% to 10.4%, n = 6) was satisfactory. The derivatized PA remained stable within 48 h. The limit of detection and the limit of quantification could reach 2 μg/kg and 50 μg/kg, respectively. As a result, the method was successfully applied to detect PA in over 42 batches of the red yeast rice samples. It indicated a low risk of PA contaminations in the red yeast rice products made in China. Furthermore, its application to the other health food products containing red yeast rice demonstrated the applicability of the established method.

## 1. Introduction

Red yeast rice is a traditional Chinese medicine produced by fermenting rice with *Monascus* species, which is also widely used as a functional food [1,2]. It is rich in natural pigments, such as monascin, ankaflavin, etc., and possesses various bioactive compounds, including monacolins as representative ingredients, alkaloids, steroids, terpenoids, flavonoids, and polyphenols, among others [2,3,4,5]. Traditional applications in clinical settings and some modern pharmacological studies have demonstrated that red yeast rice is effective for the treatment and prevention of metabolic diseases [1,6,7,8]. In the food industry, red yeast rice is highly favored by consumers for its coloring and preservation properties. In the pharmaceutical field, it is well known that monacolin K is a natural HMG-CoA reductase inhibitor and is widely used for preventing and treating hypercholesterolemia [9,10]. In recent years, with growing health awareness and increasing demand for natural and healthy products, more countries and regions have started to accept and promote red yeast rice products.

However, the recent detection of puberulic acid (PA) in red yeast rice products has drawn significant attention due to its potent renal toxicity. PA is a secondary metabolite produced by *Penicillium* species, and it has been reported to have antimalarial activity [11,12]. In early 2024, high concentrations of PA were found in the red yeast rice dietary supplements manufactured by Kobayashi Pharmaceutical Company in Japan, leading to renal impairment in several consumers and sparking international concern [13]. This incident not only poses a serious threat to consumer health but also dealt a significant blow to the market reputation of red yeast rice products. It is highly desirable to have reliable detection and determination of PA in red yeast rice products for understanding their potential safety risk. However, to the best of our knowledge, there are currently no published reports on detection methods specifically designed for PA. Drawing from chromatographic analyses of its structurally similar compound hinokitiol [14,15,16], which exhibits chelating activity of the tropolone ring, instability to heat, and adsorption on the stationary phase, these are likely to pose substantial challenges in developing a robust detection method for PA, which also possesses a tropolone framework. Indeed, our preliminary studies demonstrated that these characteristics significantly complicated both the extraction and detection processes. Therefore, there is an urgent need to comprehensively investigate and establish an efficient and sensitive detection method for PA to ensure the safety of red yeast rice products. Currently, common methods for mycotoxin detection include high-performance liquid chromatography (HPLC) [17], liquid chromatography tandem mass spectrometry (LC-MS/MS) [18], gas chromatography tandem mass spectrometry (GC-MS/MS) [19], enzyme-linked immunosorbent assay (ELISA), and so on [20]. These methods could meet the detection requirements for most mycotoxins to a certain extent. However, due to the structural specificity of PA [21], some challenges were encountered in developing detection methods, including poor chromatographic behaviors such as the asymmetrical peak shape, weak retention on reversed-phase columns, and suboptimal MS intensity.

Derivatization techniques combined with mass spectrometry (MS) have emerged as powerful tools for the analysis of challenging compounds [22,23]. Precolumn derivatization is a common sample preparation technique in chromatographic analysis [24,25,26]. It involves chemically transforming the target compound into a derivative with different physical or chemical properties, making it more amenable to MS detection. This approach is especially useful for compounds that exhibit poor chromatographic behaviors or low ionization efficiency under standard conditions, enhancing overall analytical performance by increasing stability, improving separation, and boosting ionization efficiency. The method can be tailored to meet the specific requirements of the analysis by carefully selecting the appropriate derivatizing reagent and optimizing reaction conditions. Higashi et al. suggested employing precolumn derivatization, by labeling reactive hydroxyl group (vinyl alcohol) derived from the tropolone structure, as a means to overcome the challenges associated with the analysis of hinokitiol [14,15]. These approaches offer a valuable reference and potential strategy for developing detection methods for PA, given the similar chemical properties and analytical difficulties between these compounds.

In the present work, a systematical study was conducted for detection and quantification of PA in red yeast rice. A LC-MS/MS method integrated with the precolumn derivatization was developed for the first time. Methylation using trimethylsilyldiazomethane (TMSCHN_2_) was applied to enhance the analytical performance of PA, including its stability, chromatographic peak shape, retention, and ionization efficiency. To pursue the sensitivity and reliability of detection, a compound-specific multiple reaction monitoring (MRM) scan based on LC-MS/MS was utilized to overcome interferences from ingredients inherent in red yeast rice. This study not only contributes to ensuring the safety of red yeast rice products but also provides effective technical support to relevant regulatory authorities. Through systematic research and method optimization, we aimed to achieve breakthroughs in PA detection in red yeast rice products, offering new ideas and technological solutions for future food safety and quality control.

## 2. Results and Discussion

### 2.1. Chromatographic Behavior

**Figure 1:**
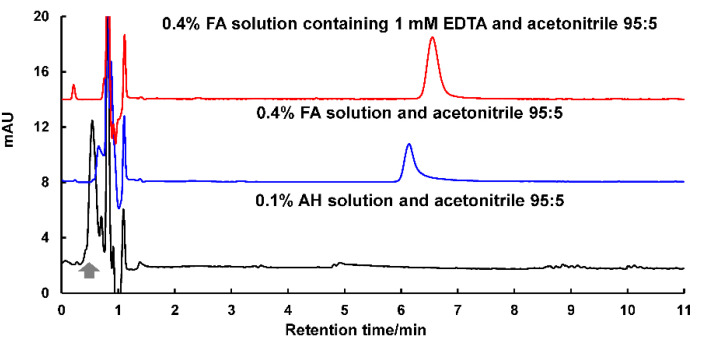
HPLC separation of PA detected at 270 nm with different chromatographic additives.

The unique structure of PA features a tropolone framework substituted with two additional hydroxyl groups and one carboxyl group. Due to the keto-enol tautomerization, PA naturally exists in two possible structural forms, although the equilibrium between these forms is not well understood. On the other hand, coordination between PA and metal ions has been reported in relevant studies on tropolone [15,21]. Our preliminary analysis showed that the chromatographic separation of PA presented a single peak with significant tailing at 270 nm. Especially at low concentration levels, PA is almost completely adsorbed by the liquid chromatography system, leading to its peak being submerged in the baseline. Even when using a low-adsorption liquid chromatographic system, such as the commercial ACQUITY Premier system from Waters, the desired peak shape could not be achieved. As illustrated in Figure 1, with a reversed-phase CAPCELL PAK C18 MG column, which has low metal impurity content and reduced residual silanol groups, almost no chromatographic retention of PA presented by using an alkaline mobile phase composed of 0.1% AH solution and acetonitrile (95:5). Although the addition of an acidic chromatographic additive by replacing 0.1% AH with 0.4% FA could enhance retention, peak tailing still persisted. To address this issue, ethylenediaminetetraacetic acid (EDTA) with the concentration of 1 mM was specifically added to the acidic mobile phase (0.4% FA solution) to compete for coordination with any residual metal ions in the separation system. As a result, the chromatographic peak shape of PA was markedly improved. This enhancement allowed for the detection of a PA standard solution with a concentration above 1 μg/mL. Nevertheless, its application to the complex matrix of red yeast rice still faces challenges due to interferences from the inherent ingredients. Its detection sensitivity at mg/kg levels is far from meeting the monitoring requirements. A MRM scan based on a triple quadrupole (QQQ) mass spectrometry has been considered the “gold standard” for qualitative and quantitative analyses of trace-level targets, offering superior sensitivity and selectivity [27]. However, the high residue of EDTA in the mass spectrometry system limits the transfer of this HPLC method to an LC-MS system, thereby hindering the pursuit of higher detection sensitivity and selectivity.

### 2.2. Precolumn Derivatization

**Figure 2:**
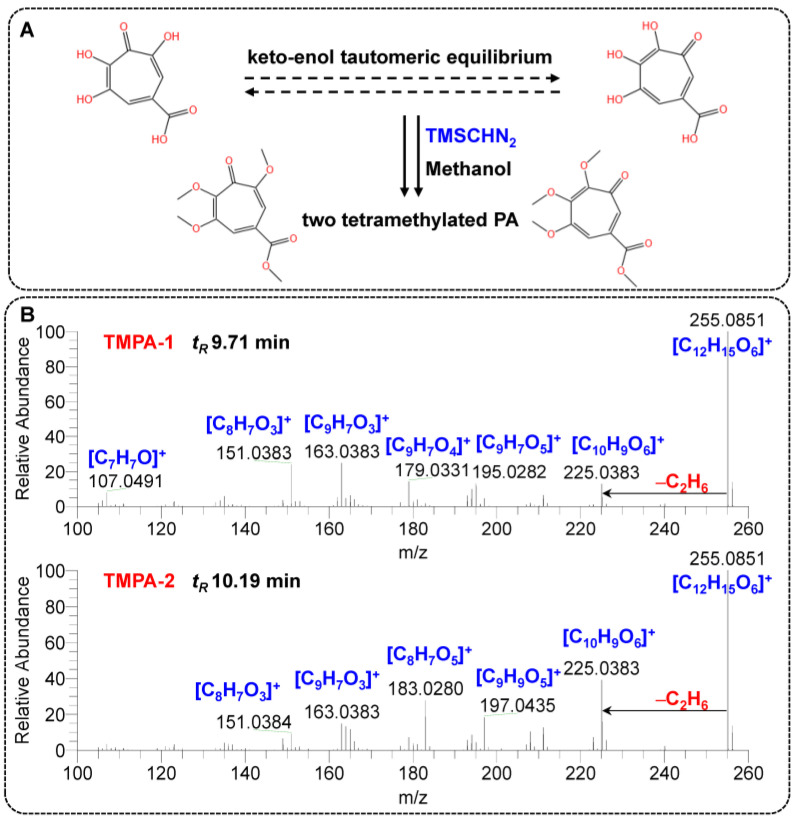
Structures of PA and its methylated products (**A**) and high-resolution ddMS^2^ spectra of the TMPAs and fragments annotating (**B**).

To address the aforementioned issues encountered in preliminary analyses, the study systematically investigated the derivatization possibilities of PA. As a result, methylation with a safe and stable commercial reagent, TMSCHN_2_, was selected. Referring to the existing literature on similar reactions [26,28,29,30], methanol, due to environmental and safety considerations, was used as the methylation reaction medium. As a result, due to the keto-enol tautomeric equilibrium of PA, two tetramethylated forms of PA were ultimately generated, which were separately named as TMPA-1 and TMPA-2 (Figure 2A). As depicted in Figure 2B, the products were confirmed with liquid chromatography tandem high-resolution Q-Orbitrap mass spectrometry. The fragments of TMPA-1 and TMPA-2 were annotated based on their high-resolution ddMS^2^ spectra. A significant difference was observed in the collision-induced dissociation of the precursor ions at *m*/*z* 255.0863 ([C_12_H_15_O_6_]^+^). Unfortunately, it is difficult to deduce the proposed fragmentation pathways and differentiate them solely based on the available MS data. Their unambiguous identification would require additional NMR analyses, which are outside the scope of this study.

**Figure 3:**
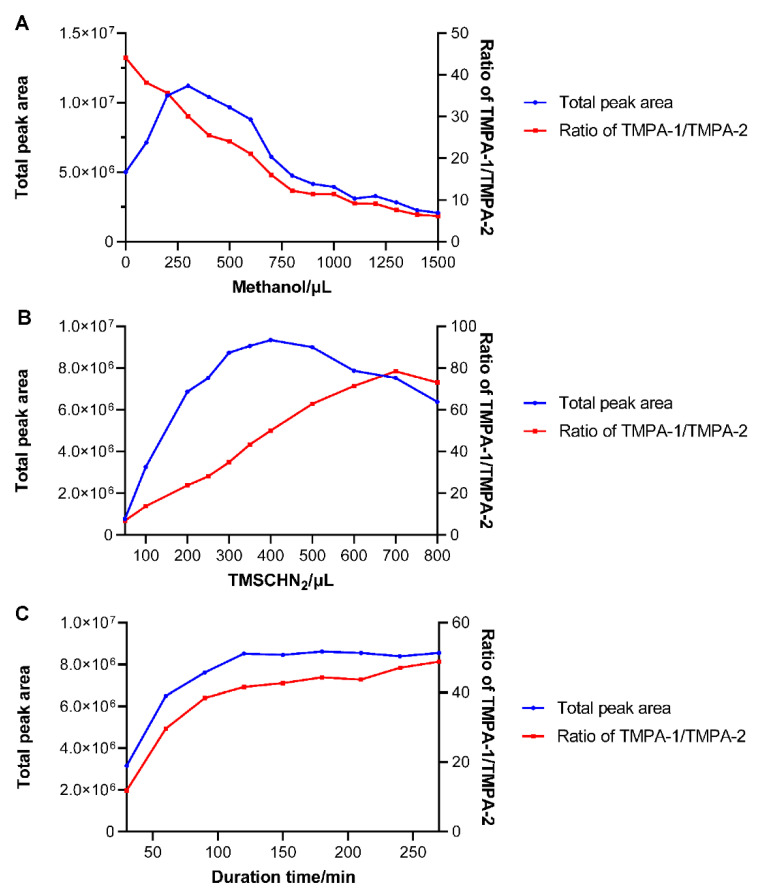
Optimization of methylation conditions including methanol volume (**A**), dosage of TMSCHN_2_ (**B**), and reaction time (**C**) with a standard solution of 5 μg/mL diluted with the methanol extract of blank red yeast rice.

To realize the methylation efficiency of PA, the volume of methanol as the reaction solvent, the dosage of TMSCHN_2_ as the methylated reagent, as well as the reaction time needed were individually examined. Considering the influence of the inherent ingredients in red yeast rice, a standard solution of 5 μg/mL diluted with the methanol extract of blank red yeast rice was used. As depicted in Figure 3A, with a 300 μL volume of TMSCHN_2_, the total peak area of the two methylated products were measured for various volumes of methanol ranging from 0 μL to 1500 μL. The product yield initially increased and then decreased with the increase in methanol in the reaction system. And the maximum yield was obtained by adding 300 μL methanol. In addition, the area ratio of TMPA-1 to TMPA-2 was also investigated, revealing a remarkable decreasing trend with the increase in methanol. Subsequently, the total peak area of the two products was determined by varying the TMSCHN_2_ volume from 50 μL to 800 μL. As shown in Figure 3B, the product amount exhibited an initial increasing trend followed by a slight decrease, with the peak value achieved at 400 μL of TMSCHN_2_. Moreover, the area ratio of TMPA-1 to TMPA-2 showed an increasing trend. It was speculated that the polarity change of the reaction solvent, due to mixing different volumes of methanol and hexane, influenced the keto-enol tautomeric equilibrium of PA, thus resulting in the different yields of the two products. Furthermore, Figure 3C showed the test of the reaction time, indicating that the methylation of PA was substantially completed within 2 h. The area ratio of the products also tended to stabilize after 2 h. Therefore, this experiment determined the critical conditions for the derivatization of PA.

### 2.3. Method Development

Due to the unavailability of pure reference substances for direct infusion, chromatographic baseline separation is necessary for establishing a compound-specific data acquisition method. The methylated products were initially analyzed in both positive and negative ion modes, revealing that their response ([M + H]^+^) in the positive mode was significantly higher than that in the negative mode. Hence, the decision was made to establish the LC-MS/MS method in the positive ion mode. To determine the ion transitions of MRM scan, the targeted product scan of *m*/*z* 255.1 was performed by separately setting collision energies as 10 eV, 30 eV, and 50 eV. The fragments with high abundance and common to both methylated products were prioritized for selection, including *m*/*z* 225.0, *m*/*z* 211.1, *m*/*z* 194.1, *m*/*z* 179.0, and *m*/*z* 163.0. Their optimal collision energy and declustering voltage settings were subsequently determined. Moreover, for chromatographic separation, the elution gradient of the mobile phase was carefully optimized to avoid co-eluting with the intrinsic interference ions from the red yeast rice. Taking both MS intensity and matrix interferences into consideration, the ion transitions of *m*/*z* 255.1/194.1 and *m*/*z* 255.1/225.0 were ultimately determined for the purpose of quantitation and qualification, respectively. Their collision energies were separately set at 26 eV and 30 eV and the declustering potential at 100 V.

**Figure 4:**
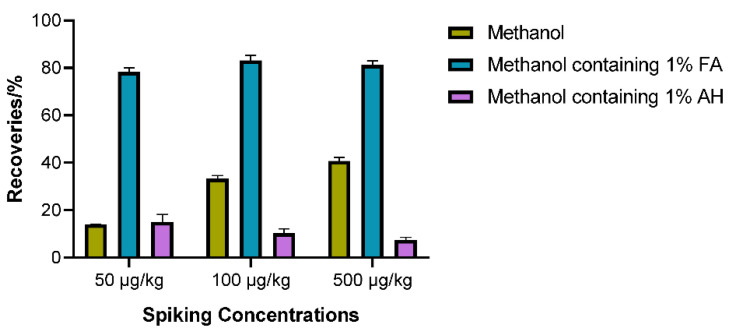
Effect of extraction solvent on recoveries of PA by spiking blank red yeast rice sample at three different levels (50, 100, and 500 μg/kg) in triplicate.

Furthermore, the extraction solvent was tested for sample preparation. A simple shaking method was applied, and the recoveries of PA at three spiking levels of 50, 100, 500 μg/kg in the blank red yeast rice sample were assessed. Taking into account both the extraction procedure of PA and the requirements of the methylation reaction medium, methanol was initially chosen as the extraction solvent in this experiment. However, it was found that the recoveries of PA at three spiking levels were only 14.1% to 40.7%. Subsequently, formic acid and ammonium hydroxide solution were then separately added into methanol to improve extraction efficiency. As seen in Figure 4, when using a methanol solution containing 1% formic acid, recovery rates ranging from 78.4% to 83.1% were obtained, which met the recovery requirement between 70 and 120%. On the other hand, to investigate the potential influence of 1% formic acid methanol matrix on the methylation reaction, we conducted a comparative analysis of the total peak areas and their area ratios for the two methylated products under two different extraction solvents. The standard solutions of 5 μg/mL separately diluted with the methanol and 1% formic acid methanol extracts of blank red yeast rice were analyzed under the optimized derivatization conditions. As given in Table S1, the total peak areas of the methylated products obtained with the two matrix standard solutions were consistent. Although there were slight differences in the area ratios of TMPA-1 (at 11.0 min) to TMPA-2 (at 14.5 min), the product of TMPA-1 was predominant in both cases. The overall consistency of the results indicated that the utilization of 1% formic acid methanol did not significantly alter the yield of the methylation reaction profile. Consequently, it was finally decided to use a 1% formic acid methanol solution as the extraction solvent.

### 2.4. Method Performance

**Figure 5:**
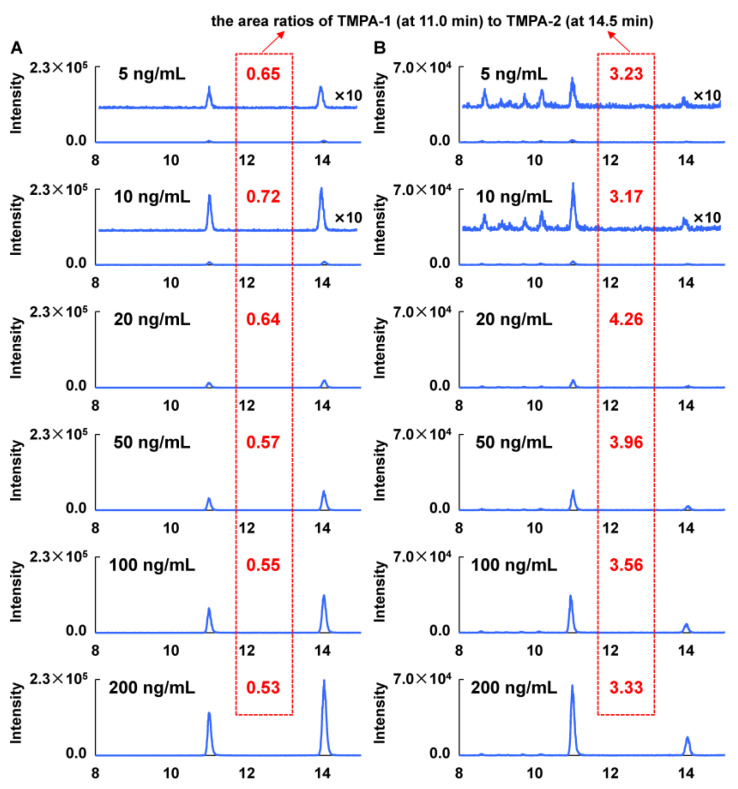
The extracted ion chromatograms (the channel of *m*/*z* 255.1/225.0) of the methylated PA at the concentrations of 5, 10, 20, 50, 100, and 200 ng/mL, including the solvent reference solutions (**A**) and the matrix reference solutions (**B**).

Regarding the detection of PA in trace levels, the method validation was carried out referring to the European Union Reference Laboratories for Residues of Pesticides (2021) [31]. To ensure the determination accuracy with the LC-MS/MS method integrated with the precolumn derivatization, the matrix effect was initially assessed. A series of reference solutions at the concentrations of 5, 10, 20, 50, 100, and 200 ng/mL were prepared separately using the extraction solvent and the extracted matrix solution. By the precolumn derivatization and the LC-MS/MS analyses, the two calibration curves were measured by the linear regression between the concentrations of PA and the total area of the two products (1/X weighted). According to the slopes of the two standard curves, the matrix effect was calculated to be –65%, indicating a significant ion suppression effect. It was also found that the area ratios of the TMPA-1 (at 11.0 min) to TMPA-2 (at 14.5 min) determined in the matrix reference solutions were different from that in the solvent reference solutions, which were separately calculated as 3.17–4.26 and 0.53–0.72 based on the acquired area in the channel of *m*/*z* 255.1/225.0. As given in Figure 5, in comparison, the TMPA-1 proportion yielded in the matrix reference solutions was remarkably higher than that in the solvent reference solutions, about 6-fold as assessed by the area ratios of TMPA-1 (at 11.0 min) to TMPA-2 (at 14.5 min) (0.53–0.72 vs. 3.17–4.26). Moreover, for both the solvent and matrix reference solutions, there was a slight difference in the area ratios under different concentration levels. This demonstrates that the matrix also has a certain influence on the keto-enol tautomeric equilibrium of PA. Although the TMPA-1 was dominant in the chromatogram of channel *m*/*z* 255.1/194.1 (Figure S1), it was necessary to apply the sum of two peak areas for quantification purpose. Thus, to compensate for the matrix effect, a matrix-matched standard calibration curve was ultimately utilized.

The method linearity and range were evaluated by the matrix-matched calibration curves constructed in triplicate on individual days. As given in Table 1, the correlation coefficients (*r*) in the range from 5 to 200 ng/mL were all greater than 0.999, indicating an excellent linearity. The recovery tests were carried out by spiking PA into the negative red yeast rice sample at levels of 50, 100, and 500 μg/kg in six replicates. As shown in Table 2, the average recoveries at three levels ranged from 82.2% to 84.2%, meeting the requirement between 70 and 120%, which suggests an acceptable trueness of the method. The precision was confirmed with the RSDs of 2.1–10.4%. For reproducibility, intra-day and inter-day variability assays presented RSDs at three spiking levels less than the validation requirement of below 20%. The stability testing suggests that the methylated PA could remain stable within at least 48 h. This proves that the methylation derivatization also plays an important role in improving the stability of PA. Referring to the spiking level meeting the criteria for recovery and precision, the LOQ was determined as 50 μg/kg. By diluting the matrix reference solution to afford an S/N of 3, the LOD was calculated as 2 μg/kg. The sensitivity of the developed LC-MS/MS method is significantly higher than that of the HPLC method.

In conclusion, the methylation derivatization of PA showed a noticeable enhancement of analytical performance from the following aspects: (1) stabilizing the keto-enol tautomeric equilibrium, thus increasing the stability of the target; (2) impeding coordination with metal ions, resulting in an optimal chromatographic peak shape; (3) achieving the strong retention on reversed-phased column by decreasing the polarity of the target; and (4) increasing detection intensity by ionization efficiency improvement.

### 2.5. Application to Real Samples

In order to evaluate the applicability of the established method, the analyses of 42 batches of red yeast rice samples collected from different companies in China were performed. As a result, the positive detection of PA was not found for these samples. It indicated a low risk of PA contamination in the red yeast rice products made in China. In addition, the method was also utilized to analyze health products containing red yeast rice, such as Nattokinase health supplements. Regarding the more complex matrix, sensitivity with an LOD of 2 μg/kg could be achieved, and no positive detection of PA was found. Hence, it is promising that the application of the presented LC-MS/MS method integrating precolumn derivatization could be extended to other related products with a possible infection risk of *Penicillium* for detection of PA.

## 3. Conclusions

In the present work, we conducted a systematic investigation on the detection of PA. An HPLC method using the 0.4% formic acid solution containing 1 mmol/L EDTA and acetonitrile as the mobile phase was established for analysis of PA. Considering the limited sensitivity of this method, especially applied to complex red yeast samples, a simple and sensitive method based on LC-MS/MS and precolumn derivatization is herein proposed. The methylation procedure of PA with TMSCHN_2_ and the MRM data acquisition were comprehensively optimized to enhance detection sensitivity and selectivity. The assessment of key operational and analytical parameters, including linearity, trueness, precision, and sensitivity, demonstrated that the LC-MS/MS method performance could meet the validation criteria of the related guidelines of SANTE/11312/2021. Ultimately, the method was successfully applied to the real red yeast rice samples and the related health products. The results demonstrated the proposed approach is practical for the detection of PA. Specially, the methylating derivatization overcomes the bottleneck in the detection method development of PA, facilitating sensitivity enhancement.

## 4. Materials and Methods

### 4.1. Chemicals and Reagents

The analytical standard of PA (0.1 mg; lot, 2418062; purity, 98.7%) used in this study was purchased from ANPEL-TRACE Standard Technical Services (Shanghai, China) Co., Ltd. Acetonitrile with LC-MS grade purchased from Merck KGaA (Merck, Darmstadt, Germany) and ultra-pure water prepared by a Millipore Alpha-Q water purification system (Millipore, Bedford, MA, USA) were used for chromatographic separation. Formic acid (Thermo Fisher Scientific, Shanghai, China; FA), ammonium hydroxide solution (~10% in H_2_O, Sigma-Aldrich, St. Louis, MO, USA; AH), and 10 M ammonium formate (Sigma-Aldrich Co., St. Louis, MO, USA; AF) of LC/MS grade were used as chromatographic additives. Analytical methanol and formic acid (Shanghai Ling feng Chemical Reagent Co., Ltd., Shanghai, China) were used for sample preparation. Trimethylsilyldiazomethane (2.0 mol/L in hexane, TMSCHN_2_) for methylation of PA was purchased from Aladdin (Shanghai, China).

A total of 40 batches of red yeast rice samples were collected from 31 enterprises, and the detailed information is provided in Table S2. These samples were crushed into powders and stored at −20 °C before analysis.

### 4.2. Preparation of Standard Solutions

The stock solution of PA standard was prepared by dissolving the accurately weighed 0.1 mg powders (calibration factor, 98.7%) with methanol. A standard solution of 100 μg/mL was obtained and then stored at −20 °C, avoiding light. Working standard solutions at the concentrations of 5, 10, 20, 50, 100, and 200 ng/mL were obtained by diluting the stock solution with methanol containing 1% formic acid and the test solution of blank red yeast rice sample, respectively.

### 4.3. Sample Preparation

An aliquot of 1.0 g sample powders were accurately weighed into a 50 mL centrifuge tube. Shaking extraction with 10 mL methanol containing 1% formic acid for 30 min was performed. After centrifugation of the extract at 4000 rpm for 10 min, the test solution was obtained. Then, 100 μL of the test solution was utilized for derivatization. Methanol of 300 μL as the reaction solvent was added, and the optimized volume of TMSCHN_2_ was 400 μL. After vortexing, the mixed solution was placed at room temperature for reaction. After 2 h, the solution was concentrated to remove hexane under a gentle stream of nitrogen and then was diluted to 1 mL with methanol.

### 4.4. HPLC Condition

An Agilent 1290 series UPLC system (Agilent, Waldbronn, Germany) equipped with a binary solvent system, an auto-sampler, a column manager, and a DAD detector was applied. A CAPCELL PAK C18 MG (S-2) (2.0 × 150 mm, 2.7 μm) column was applied. The isocratic elution with the mobile phase of 0.4% FA solution containing 1 mmol/L EDTA and acetonitrile (95:5, *v*/*v*) was conducted at the flow rate of 0.3 mL/min. The column temperature was set at 30 °C. The DAD detector was set at 270 nm. The injection volume was 2 μL.

### 4.5. HPLC-MS/MS Analysis

An Agilent 1260 series HPLC system (Agilent, Waldbronn, Germany) coupled to a triple quadrupole mass spectrometer system (5500, AB Sciex Instruments, Concord, ON, Canada) by an electrospray ion (ESI) source was used for data acquisition. Chromatographic separation was conducted on an Agilent Poroshell EC-C18 column (3.0 × 150 mm, 2.7 μm). The mobile phase system consisted of water (A) and methanol (B), both containing 0.4% FA and 2 mmol/L AF (*v*/*v*). The gradient elution program was followed as below: 0–2 min, 20% (B); 2–5 min, 20–30% (B); 5–13 min, 30–35% (B); 13–14 min, 35–95% (B); 14–17 min, 95% (B). The flow rate was set as 0.45 mL/min. The column was maintained at 30 °C. The injection volume was 1 μL.

A multiple reaction monitoring (MRM) scan in the positive mode was utilized for data acquisition. The ESI source parameters were set as follows: curtain gas (CUR) of 40 psi, collision gas (CAD) of 7 psi, ion spray voltage (IS) of 4500 V, temperature (TEM) of 450 °C, nebulizer gas (GS1) of 50 psi, heater gas (GS2) of 50 psi, entrance potential of 10 V, and collision cell exit potential (CXP) of 14 V. The precursor ion was *m*/*z* 255.1 ([M + H]^+^). The product ions of *m*/*z* 194.1 and *m*/*z* 225.0 were separately selected as the quantitative and qualitative ions. The optimized collision energies were 26 V and 30 V, respectively. The declustering potential was 100 V. The software of Analyst 1.5.1 and MultiQuant 2.1.1 (AB Sciex Instruments, Concord, ON, Canada) were separately applied for data acquisition and processing.

### 4.6. Method Validation

The method was validated referring to the European Union Reference Laboratories for Residues of Pesticides (2021) [31]. The parameters including matrix effect, linearity of the calibration curve, accuracy, precision, and sensitivity were evaluated. By separately measuring the calibration curves in the blank matrix and in the 1% formic acid methanol solution, the matrix effect (%) was determined by the equation of [slope of the matrix calibration curve/slope of the solvent calibration curve-1] × 100%. When the ionization suppression or enhancement effect is severe, a matrix-matched standard calibration curve is required to compensate for the matrix effect [31]. The linear-regression correlation coefficient (*r*) of the calibration curve at six concentration levels (5, 10, 20, 50, 100, and 200 ng/mL) was calculated to estimate the method linearity. The accuracy (as % recovery) and precision (as % RSD, n = 6) were assessed by three spiking levels with six replicates (n = 6) in the recovery experiments. Reproducibility was expressed as intra-day and inter-day precisions by replicated spiking experiments. The matrix reference solution after derivatization was deposited at room temperature for 48 h to evaluate the stability of the methylated PA. The limit of quantification (LOD) refers to the lowest spiking level meeting the criteria for recovery (recovery, 70–120%) and precision (RSD ≤ 20%). The limit of detection (LOD) was determined by diluting the matrix reference solution to provide a signal-to-noise ratio (S/N) of 3.

## Figures and Tables

**Table 1:** Linearity results obtained from three independent runs over consecutive days.

Days	Calibration Equation	Correlation Coefficient (*r*)
1	y = 2731.96258x + 4430.29628	0.99952
2	y = 2856.24563x + 3307.12670	0.99959
3	y = 2763.40498x + 3903.81372	0.99974

**Table 2:** Recoveries and RSDs (n = 6) by spiking blank red yeast rice sample at three different levels (50, 100, and 500 μg/kg) in six replicates.

No.	Spiking Level (μg/kg)	Recovery (%)	Average (%)	RSD (%)
1	50	81.865	84.2	10.4
2	50	73.081		
3	50	84.404		
4	50	99.906		
5	50	84.583		
6	50	81.388		
7	100	84.315	82.2	3.6
8	100	83.727		
9	100	85.802		
10	100	81.904		
11	100	79.730		
12	100	78.011		
13	500	86.515	83.7	2.1
14	500	84.248		
15	500	84.099		
16	500	82.383		
17	500	83.050		
18	500	81.652		

## Data Availability

The original contributions presented in this study are included in the article/Supplementary Material. Further inquiries can be directed to the corresponding authors.

## Supplementary Materials

The following supporting information can be downloaded at https://www.mdpi.com/article/10.3390/toxins17010011/s1: Table S1: Comparison of the total peak areas and their area ratios for the two methylated products under two different extraction solvents; Table S2: Information on 42 batches of the red yeast rice samples and two health supplements; Figure S1: The extracted ion chromatograms of the solvent and matrix reference solutions at the concentration of 50 ng/mL (channel of *m/z* 255.1/194.1).

## Abbreviations

PA: puberulic acid; TMPA, tetramethylated puberulic acid; EDTA, ethylenediaminetetraacetic acid; TMSCHN_2_, trimethylsilyldiazomethane; FA, formic acid; AH, ammonium hydroxide solution; AF, ammonium formate.
