# Supplementary material for: Determination of Puberulic Acid in Monascus-Fermented Red Yeast Rice by LC-MS/MS Combined with Precolumn Derivatization
Source: Toxins (Basel). 2024 Dec 29;17(1):11. doi: 10.3390/toxins17010011 (PMC11769100; doi:10.3390/toxins17010011)
Supplement: Supplementary file 1 [file toxins-17-00011-s001.zip › toxins-3372980-supplementary.pdf]

<Toxins>

Determination of puberulic acid in Monascus-fermented red yeast rice by LC-MS/MS combined with precolumn derivatization

Hui-Qin Pan [1,†], Rui Feng [1,†], Yan-Nan Tan [1], Xiao-Ya Qin [1,2], Yi-Min Cao [1], Xiu-Hong Mao [1], Qing Hu [1,*], and Heng Zhou [1,*]

1 NMPA Key Laboratory for Quality Control of Traditional Chinese Medicine, Shanghai Institute for Food and Drug Control, 1500 Zhangheng Road, Shanghai 201203, China;

2 School of pharmacy, Shanghai University of Traditional Chinese Medicine, 1200 Cailun Road, Shanghai 201203, China

* Correspondence: huqingyjs@163.com (Q.H.); zhouheng@sifdc.org.cn (H.Z.)

† These authors contributed equally to this work.

Table S1 Comparison of the total peak areas and their area ratios for the two methylated products under two different extraction solvents.

	Total peak area of TMPA-1 and TMPA-2	Average	Area ratio of TMPA-1/TMPA-2	Average
**mathanol matrix**	9.13E+06	9.17E+06	27.7	25.5
	9.21E+06		23.3	
**1% formic acid mathanol matrix**	9.25E+06	9.23E+06	31.1	26.4
	9.21E+06		21.8	

Table S2 Information on 42 batches of the red yeast rice samples and two health supplements.

No.	Collection regions	Lot No.
1	Anhui Province, China	20220402
2	Zhejiang Province, China	220811
3	Sichuan Province, China	/
4	Hebei Province, China	221217
5	Fujian Province, China	231101
6	Hebei Province, China	16623016
7	Hebei Province, China	C653230401
8	Sichuan Province, China	230604
9	Unknown	/
10	Anhui Province, China	231001
11	Zhejiang Province, China	B23111401-01
12	Anhui Province, China	230703
13	Hebei Province, China	2309001
14	Sichuan Province, China	20230601
15	Sichuan Province, China	230602
16	Guangdong Province, China	C202312001
17	Zhejiang Province, China	20260614
18	Heilongjiang Province, China	23090401
19	Fujian Province, China	/
20	Henan Province, China	230402
21	Unknown	/
22	Fujian Province, China	/
23	Fujian Province, China	/
24	Sichuan Province, China	/
25	Unknown	/
26	Anhui Province, China	/
27	Zhejiang Province, China	231201
28	Fujian Province, China	/
29	Unknown	/
30	Sichuan Province, China	230901
31	Sichuan Province, China	231003
32	Sichuan Province, China	231004
33	Sichuan Province, China	240402
34	Unknown	20230906
35	Unknown	20240104
36	Unknown	20240314
37	Unknown	20240315
38	Unknown	24011715
39	Unknown	/
40	Unknown	/
41	Unknown	/
42	Unknown	2024031602
	health supplements	
S1	NATTOKINASE	23Y0511-2
S2	NATTOKINASE 4000FU	NAL24Y02

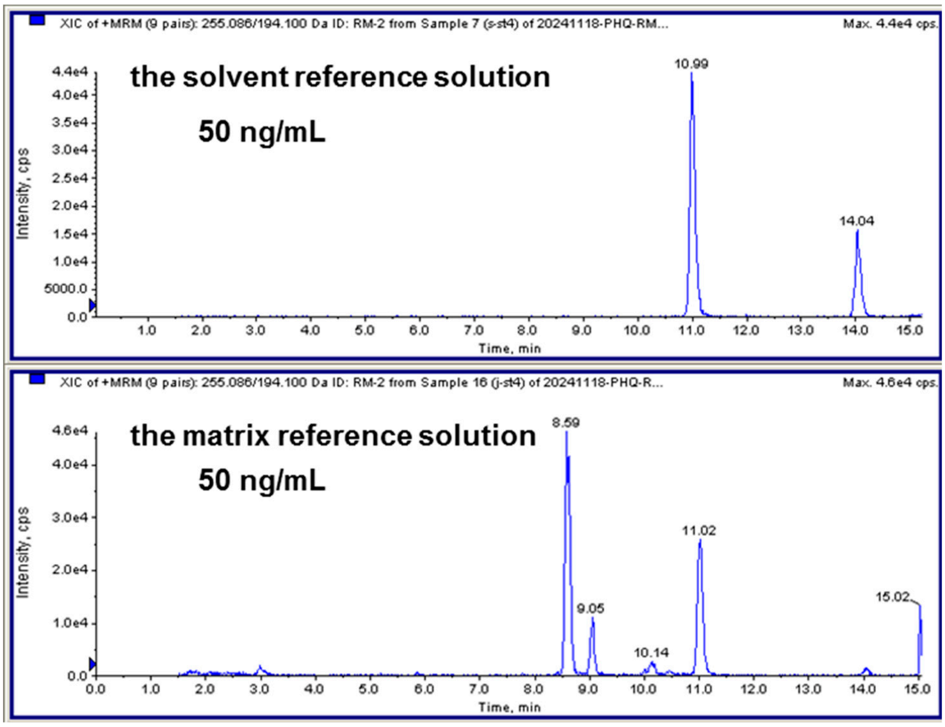

Figure S1. The extracted ion chromatograms of the solvent and matrix reference solutions at the concentration of 50 ng/mL (channel of *m/z* 255.1/194.1).
